# Metabolic Syndrome Is Independently Associated with Pain Severity in Psoriatic Arthritis

**DOI:** 10.3390/biomedicines14081752

**Published:** 2026-08-04

**Authors:** Ludovica Lamberti, Damiano Currado, Francesca Saracino, Francesca Trunfio, Leonardo Frascà, Annalisa Marino, Marta Vomero, Irene Genovali, Sebastiano Lorusso, Erika Corberi, Matteo Padovan, Guglielmo Giannini, Lyubomyra Kun, Onorina Berardicurti, Roberto Giacomelli, Luca Navarini

**Affiliations:** 1 Rheumatology and Clinical Immunology, Department of Medicine, School of Medicine, University of Rome Campus Biomedico, 00128 Rome, Italyleonardo.frasca@unicampus.it (L.F.); matteo.padovan@unicampus.it (M.P.); l.navarini@policlinicocampus.it (L.N.); 2 Clinical and Research Section of Rheumatology and Clinical Immunology, Fondazione Policlinico Universitario Campus Bio-Medico, Via Alvaro del Portillo 200, 00128 Rome, Italy; irene.genovali@policlinicocampus.it (I.G.); lyubomyra.kun@policlinicocampus.it (L.K.);; 3 Department of Life, Health and Environmental Sciences, University of L’Aquila, Via Vetoio SNC, Ed. Camillo De Meis, 67100 L’Aquila, Italy; 4 Rheumatology, Department Life Sciences, Health and Health Professions, Link Campus University, 00165 Roma, Italy

**Keywords:** metabolic syndrome, psoriatic arthritis, brain

## Abstract

**Objective:** Pain in psoriatic arthritis (PsA) is multifactorial and may persist despite apparently controlled inflammatory activity. Metabolic syndrome (MetS), a frequent comorbidity in PsA, could contribute to increased pain burden beyond inflammatory disease activity. The objective was to investigate the association between MetS and pain burden in PsA, focusing on pain intensity (BPI severity; primary outcome) and pain-related functional impact (BPI interference; secondary outcome), and to assess whether these associations are independent of disease activity (DAPSA) and fibromyalgia. **Methods:** PSABRAIN is a cross-sectional, monocentric study including consecutive adult patients with PsA (CASPAR criteria). MetS was defined according to modified ATP III criteria. Pain outcomes were assessed using the Brief Pain Inventory (BPI) severity and interference subscales. Group comparisons (MetS vs. no MetS) were performed using appropriate parametric/non-parametric tests. Univariable and multivariable linear regression models were fitted for BPI severity and BPI interference. Multivariable models were adjusted for age, sex, disease duration, fibromyalgia, and DAPSA. Robustness analyses replaced MetS with BMI (continuous), obesity, and overweight. **Results:** A total of 187 PsA patients were included (44 with MetS; 143 without). MetS patients were older (median 62 vs. 57 years, *p* = 0.0017), had higher BMI (median 30.1 vs. 25.5 kg/m^2^, *p* < 0.001) and a higher prevalence of obesity (56.8% vs. 15.4%, *p* < 0.001), while DAPSA did not differ between groups (*p* = 0.819). MetS patients reported higher BPI severity (median 5.75 vs. 4.5, *p* = 0.038) and BPI interference (median 6.86 vs. 5.5, *p* = 0.044). In univariable regression, MetS was associated with higher BPI severity (β = 0.93, 95% CI 0.08 to 1.77; *p* = 0.032) but not with BPI interference (β = 0.81, 95% CI −0.13 to 1.75; *p* = 0.091). In adjusted models, MetS remained independently associated with BPI severity (β = 0.99, 95% CI 0.23 to 1.75; *p* = 0.011), whereas its association with BPI interference was not significant (β = 0.69, 95% CI −0.20 to 1.57; *p* = 0.126). In alternative models, BMI and obesity were independently associated with both outcomes (BPI severity: BMI β = 0.097/kg/m^2^, *p* = 0.004; obesity β = 0.91, *p* = 0.010; BPI interference: BMI β = 0.095/kg/m^2^, *p* = 0.015; obesity β = 1.22, *p* = 0.003). **Conclusions:** In PsA, MetS is independently associated with higher pain severity but not with pain interference after adjustment for disease activity and fibromyalgia. Adiposity measures show consistent independent associations, particularly with pain interference, supporting the relevance of metabolic and weight-related factors in the multidimensional assessment of pain in PsA.

## 1. Introduction

Psoriatic arthritis (PsA) is a chronic inflammatory musculoskeletal disease characterized by a heterogeneous spectrum of manifestations, including peripheral arthritis, axial involvement, enthesitis, dactylitis, and psoriasis affecting the skin and nails [1,2,3]. Despite improvements in the management of PsA with conventional and targeted therapies, a substantial proportion of patients continue to experience persistent pain, impaired function, and reduced quality of life, even when objective inflammatory indices appear controlled [4,5,6,7,8]. This discrepancy highlights the complexity of pain mechanisms in PsA and the need for a multidimensional assessment beyond traditional disease activity scores.

Metabolic syndrome (MetS) is a highly prevalent comorbidity in PsA, with reported estimates ranging from 30% to 50% [9,10]. Defined by the clustering of central obesity, hypertension, dyslipidemia and impaired glucose metabolism, MetS contributes not only to cardiovascular risk but also to heightened systemic inflammation, altered adipokine profiles, and increased pain perception [11,12,13]. Previous studies from our group have demonstrated that, in PsA, the presence of MetS is associated with greater pain catastrophizing, worse disease activity, increased disability and reduced health-related quality of life, independent of inflammatory burden [14,15,16,17]. These findings support the hypothesis that MetS may amplify or modulate pain pathways in PsA. Importantly, pain is not a unitary construct. Pain intensity and pain-related functional interference capture distinct, clinically meaningful domains that may reflect partially different determinants and may require different management strategies. The Brief Pain Inventory (BPI) is a validated instrument that quantifies both pain severity and pain interference and has been recommended as a pragmatic approach to capture these key dimensions in clinical research [18].

Moreover, in PsA, features of abnormal pain processing are common and can substantially influence pain reporting and patient-reported outcomes, potentially confounding the relationship between comorbidities and pain burden [19]. Consistently, concomitant fibromyalgia has been reported in a substantial proportion of patients with PsA and is associated with higher patient-reported disease activity and worse symptom-related outcomes, supporting its role as a key confounder in pain analyses [20].

Therefore, in the PSABRAIN study, we investigated the association between MetS and pain burden in PsA, focusing on BPI pain severity and BPI pain interference, and assessed whether these associations were independent of disease activity (DAPSA) and fibromyalgia.

## 2. Materials and Methods

PSABRAIN is a cross-sectional, monocentric study conducted at the Rheumatology Unit of the Fondazione Policlinico Universitario Campus Bio-Medico, Rome (Italy). Adult patients (≥18 years) with a diagnosis of PsA according to the CASPAR criteria [1] were consecutively enrolled during routine outpatient visits. All participants completed a structured set of validated patient-reported outcome measures as part of standard clinimetric assessment. Patients with incomplete questionnaire data or concomitant conditions that could substantially interfere with pain interpretation (e.g., active malignancy, severe neurological disease, uncontrolled psychiatric disorders) were excluded. Metabolic syndrome (MetS) was defined according to the modified Adult Treatment Panel III criteria [21], requiring the presence of at least three of the following five components: abdominal obesity (waist circumference ≥102 cm in men and ≥88 cm in women), hypertriglyceridemia (≥150 mg/dL or lipid-lowering treatment), reduced HDL cholesterol (<40 mg/dL in men and <50 mg/dL in women), elevated blood pressure (≥130/85 mmHg or antihypertensive treatment), and impaired fasting glucose (≥100 mg/dL or antidiabetic treatment). Data were collected prospectively in an electronic database specifically designed for the study. The study received approval from the Ethics Committee of the University Campus Bio-Medico of Rome (approval No. 78.20 OSS) and was conducted in accordance with the Declaration of Helsinki and its subsequent revisions.

Clinical and demographic data were collected during routine outpatient visits and included age, sex, body mass index (BMI), disease duration, comorbidities, and current and/or previous treatments. Fibromyalgia was recorded as present/absent based on clinical diagnosis, according to the 2016 revision of the 2010/2011 fibromyalgia diagnostic criteria [22]. PsA disease activity was assessed through standard clinimetric indices, including: Tender Joint Count (TJC; 68 joints), Swollen Joint Count (SJC; 66 joints), Patient Global Assessment (PtGA; 0–10 cm VAS), Pain VAS (0–10 cm), Disease Activity in Psoriatic Arthritis (DAPSA), Psoriasis Area and Severity Index (PASI), and Leeds Enthesitis Index (LEI).

All participants completed a structured set of validated patient-reported outcome measures (PROMs), including: Brief Pain Inventory (BPI) for pain severity and interference [23], Central Sensitization Inventory (CSI) for symptoms related to central sensitization [24], painDETECT questionnaire for neuropathic-like pain features [25], Hospital Anxiety and Depression Scale (HADS) [26], Health Assessment Questionnaire (HAQ) for disability [27], EQ-5D-5L for health-related quality of life [28]. Scores for each instrument were calculated according to standard scoring procedures. All data were entered into a dedicated electronic database and checked for completeness prior to analysis. BPI severity and interference were analyzed as continuous 0–10 scores and calculated according to standard scoring procedures; severity was defined as the mean of the four pain intensity items (worst, least, average, and current pain).

Continuous variables were summarized as mean ± standard deviation (SD) or median and interquartile range (IQR), as appropriate. Categorical variables were summarized as counts and percentages. Between-group comparisons (PsA with vs. without MetS) were performed using Student’s t test or the Mann–Whitney U test for continuous variables, and the χ^2^ test or Fisher’s exact test for categorical variables, as appropriate.

Univariable linear regression models were used to explore associations between candidate predictors and BPI severity (primary outcome) and BPI interference (secondary outcome). Predictors included demographic and clinical variables (age, sex, BMI, disease duration, comorbidities, treatments), disease activity (DAPSA), fibromyalgia status, and patient-reported measures collected in the study.

To assess whether MetS was independently associated with pain outcomes, multivariable linear regression models were fitted separately for BPI severity and BPI interference. Covariates included in the multivariable models were selected a priori based on their established or potential association with pain severity in PsA and their potential role as confounders of the relationship between MetS and pain outcomes. To evaluate robustness using alternative anthropometric definitions, additional multivariable models were constructed by replacing MetS with BMI (continuous), obesity, overweight, diabetes and hypertension. Results were reported as beta coefficients (β) with 95% confidence intervals (95% CIs) and *p* values. All tests were two-tailed, and *p* < 0.05 was considered statistically significant. Statistical analyses were performed using Stata (version 14).

## 3. Results

A total of 187 patients with psoriatic arthritis (PsA) were included in the analysis: 143 without metabolic syndrome (MetS) and 44 with MetS. Patients with MetS were older (median 62 vs. 57 years, *p* = 0.0017) and had higher BMI values (median 30.1 vs. 25.5 kg/m^2^, *p* < 0.001), with a substantially higher prevalence of obesity (BMI ≥ 30 kg/m^2^) compared with patients without MetS (56.8% vs. 15.4%, *p* < 0.001). Among patients fulfilling MetS criteria, the most prevalent metabolic abnormalities were low HDL cholesterol (87.5%), hypertension (78.6%), diabetes mellitus (63.4%), hypertriglyceridemia (62.2%), and obesity (56.8%).

Disease duration was longer in the MetS group (median 7 vs. 4 years, *p* = 0.040). Inflammatory markers were higher in MetS patients (CRP and ESR, both *p* < 0.05), while composite disease activity assessed by DAPSA did not differ between groups (*p* = 0.819). Treatment exposure (conventional and biologic DMARDs) and fibromyalgia prevalence were comparable between groups (Table 1).

BPI severity was higher in the MetS group (median: 5.75 vs. 4.5, *p* = 0.038) and BPI interference was also higher (median: 6.86 vs. 5.5, *p* = 0.044). HAQ-DI was higher in MetS patients (median: 1.25 vs. 0.75, *p* = 0.029). In the EQ-5D-5L, mobility, self-care, and usual activities were worse in the MetS group (all *p* < 0.05), whereas pain/discomfort showed a borderline difference (*p* = 0.069) and anxiety/depression did not differ (*p* = 0.521). Patient-reported pain and joint pain VAS did not differ between groups (*p* = 0.117 and *p* = 0.200, respectively), and CSI total score was comparable (*p* = 0.426) (Table 1).

Univariable linear regression analyses for BPI severity are reported in Table 2. MetS was associated with higher BPI severity (β = 0.93, 95% CI 0.08 to 1.77; *p* = 0.032). Pain severity was also significantly associated with female sex (β = 1.96; *p* < 0.001), obesity (β = 1.06; *p* = 0.012), diabetes (β = 1.24; *p* = 0.006), conventional DMARD use (β = 0.77; *p* = 0.039), fibromyalgia (β = 2.40; *p* < 0.001), and higher disease activity and symptom burden, including DAPSA (β = 0.162 per point; *p* < 0.001), tender joint count (*p* = 0.004), patient-reported pain and global assessment (both *p* < 0.001), disability (HAQ; β = 1.96; *p* < 0.001), central sensitization-related symptoms (CSI; *p* < 0.001), neuropathic-like pain features (painDETECT; *p* < 0.001), mood symptoms (HADS anxiety and depression; both *p* < 0.001), and worse HRQoL indices (EQ-5D domains and EQ-VAS; all *p* ≤ 0.005). Among inflammatory markers, ESR was significantly associated with BPI severity (*p* = 0.019), whereas CRP was not (*p* = 0.141).

Univariable analyses for BPI interference are reported in Table 3. The association between MetS and BPI interference did not reach statistical significance (β = 0.81, 95% CI −0.13 to 1.75; *p* = 0.091). Interference was significantly higher in female patients (β = 2.25; *p* < 0.001) and in those with obesity (β = 1.46; *p* = 0.002), diabetes (β = 1.08; *p* = 0.033), and fibromyalgia (β = 2.43; *p* < 0.001). Higher interference was also associated with inflammatory markers (CRP and ESR; both *p* < 0.05), DAPSA (β = 0.148 per point; *p* < 0.001), pain severity (β = 0.827; *p* < 0.001), central sensitization symptoms (CSI; *p* < 0.001), neuropathic-like pain features (painDETECT; *p* < 0.001), mood symptoms (HADS anxiety and depression; both *p* < 0.001), disability (HAQ; β = 2.48; *p* < 0.001), and worse EQ-5D domain and EQ-VAS scores (all *p* < 0.001).

Multivariable linear regression models for BPI severity are presented in Table 4. After adjustment for age, sex, disease duration, fibromyalgia, and DAPSA, MetS remained independently associated with higher BPI severity (β = 0.99, 95% CI 0.23 to 1.75; *p* = 0.011). In the same model, fibromyalgia (β = 1.67; *p* < 0.001), female sex (β = 0.83; *p* = 0.029), and DAPSA (β = 0.114 per point; *p* < 0.001) were also independent correlates, whereas age and disease duration were not significant. When MetS was replaced with BMI (β = 0.097 per kg/m^2^; *p* = 0.004), obesity (β = 0.91; *p* = 0.010) and diabetes (β = 1.1; *p* = 0.005), these variables were independently associated with higher BPI severity, while overweight showed a borderline association (β = 0.65; *p* = 0.052) (Supplementary Tables S1–S4). When MetS was replaced with hypertension, hypertriglyceridemia, or low HDL cholesterol levels in separate multivariable models, none of these individual components showed a significant independent association with BPI severity.

An additional multivariable linear regression model including both MetS and BMI simultaneously were performed. In this model, neither MetS (β = 0.84, *p* = 0.064) nor BMI (β = 0.066, *p* = 0.088) remained independently associated with BPI severity. Importantly, multicollinearity was not a concern (VIF for MetS = 1.37; VIF for BMI = 1.32), suggesting that the attenuation of the associations was not driven by collinearity but rather by the shared variance between these closely related measures of metabolic status and adiposity.

In a sensitivity analysis including CSI, painDETECT, and HADS scores, MetS remained independently associated with BPI severity (β = 1.1; *p* < 0.003), while CSI was the only additional pain-related construct independently associated with the outcome (β = 0.05; *p* < 0.0001). To address the potential concern of overadjustment related to the inclusion of DAPSA, which incorporates a patient-reported pain component partially overlapping with the BPI severity outcome, we performed a sensitivity analysis replacing DAPSA with more objective inflammatory parameters (SJC and CRP). In this model, MetS remained independently associated with higher BPI severity (β = 1.13, 95% CI 0.27 to 1.98; *p* = 0.010), whereas SJC and CRP were not significant predictors, supporting the robustness of the association between MetS and pain severity beyond inflammatory activity assessed through objective measures.

Multivariable models for BPI interference are shown in Table 5. In the adjusted model including MetS, the association with BPI interference was attenuated and not statistically significant (β = 0.69, 95% CI −0.20 to 1.57; *p* = 0.126). Conversely, BMI (β = 0.095 per kg/m^2^; *p* = 0.015) and obesity (β = 1.22; *p* = 0.003) (Supplementary Tables S5 and S6) remained independently associated with greater interference in alternative models, whereas overweight was not independently associated (*p* = 0.081) (Supplementary Table S7). Across models, female sex (β ≈ 1.20–1.39; *p* ≤ 0.005), fibromyalgia (β ≈ 1.65–1.77; *p* < 0.001), and DAPSA (β ≈ 0.085–0.091 per point; *p* ≤ 0.001) were consistently associated with higher BPI interference, while age and disease duration were not.

## 4. Discussion

In this cross-sectional study of 187 patients with PsA, MetS was associated with a higher pain burden. MetS patients reported higher BPI severity and interference despite similar DAPSA, supporting the notion that pain in PsA can be dissociated from the inflammatory activity captured by composite indices [6,14,17]. In multivariable models accounting for key determinants of pain reporting (sex, fibromyalgia, and DAPSA), MetS remained independently associated with higher pain severity, whereas the association with pain interference was attenuated and no longer statistically significant. Across models, fibromyalgia and DAPSA emerged as the most consistent correlates of both BPI domains, while female sex was also independently associated, particularly with interference. In line with previous observations that metabolic comorbidity is associated with worse patient-reported outcomes in PsA, the present findings extend this concept to distinct pain domains captured by the BPI.

A clinically relevant aspect of our results is the differential behaviour of pain intensity versus pain-related functional impact. While MetS retained an independent association with BPI severity, adiposity measures (BMI and obesity) showed more consistent independent associations, particularly with interference, in robustness analyses. This pattern suggests that metabolic and weight-related factors may contribute to distinct dimensions of the pain experience. When MetS and BMI were simultaneously included in the same multivariable model, neither variable retained statistical significance, despite the absence of relevant multicollinearity. This finding suggests that the attenuation of the associations is likely related to the overlapping biological contribution of metabolic status and adiposity rather than to a statistical artefact. Given the close interrelationship between MetS components and body weight, these results should be interpreted as indicating shared metabolic and adiposity-related pathways contributing to pain burden rather than as evidence against the relevance of either factor.

Adiposity may impact daily functioning through a combination of mechanical loading, reduced physical capacity, deconditioning, and sleep disturbances, in addition to adipose tissue-driven low-grade inflammation and altered adipokine profiles. Conversely, the strong association between fibromyalgia and both outcomes reinforces the relevance of pain amplification mechanisms in PsA and highlights the importance of systematically assessing fibromyalgia when interpreting pain reports and patient-reported outcomes. In sensitivity analyses incorporating additional pain-related domains, including central sensitization, neuropathic pain features, and psychological distress, the association between MetS and pain severity remained largely unchanged. Although central sensitization showed an independent contribution to pain burden, suggesting the relevance of altered pain processing mechanisms in PsA, these findings indicate that the relationship between metabolic comorbidity and pain severity is not fully explained by pain amplification or psychological factors alone. Rather, MetS may represent an additional dimension contributing to the complexity of pain experience in PsA, potentially through interconnected inflammatory, metabolic, and functional pathways.

From a clinical perspective, these findings support integrating metabolic assessment into the evaluation of pain in PsA. Identifying MetS and obesity may help clinicians recognize patients who are at increased risk of substantial pain burden, including those with apparent discordance between inflammatory activity and symptom severity. Moreover, distinguishing pain intensity from pain-related interference may be useful for tailoring management strategies, as these domains may reflect partially different drivers. While optimizing inflammatory control remains essential, patients with prominent metabolic comorbidity and/or adiposity-related burden may benefit from broader multidisciplinary approaches targeting weight management, physical activity, sleep, and pain amplification comorbidities, alongside pharmacological treatment.

This study has limitations.

The cross-sectional design of our study represents an inherent limitation and does not allow causal relationships to be established. Although our findings suggest an independent association between MetS and pain severity, reverse causality cannot be excluded. Chronic pain and functional impairment may lead to reduced physical activity, weight gain, and subsequent metabolic alterations, potentially contributing to the development of MetS. Therefore, longitudinal studies are needed to clarify the temporal relationship between metabolic dysfunction and pain burden in patients with PsA. Furthermore, fibromyalgia was assessed according to the 2016 revision of the 2010/2011 ACR diagnostic criteria and included as a binary covariate in the multivariable models. However, because the assessment was based on the presence/absence of a fibromyalgia diagnosis rather than on a continuous evaluation of fibromyalgia-related symptom burden (e.g., Widespread Pain Index and Symptom Severity scores), residual confounding related to differences in pain severity and symptom expression among patients with fibromyalgia cannot be completely excluded.

Although the association between MetS and pain severity was statistically significant, the clinical relevance of the observed effect size should be considered. The approximately 1-point increase in BPI severity associated with MetS is within the range of changes that have been proposed as potentially clinically meaningful in pain assessment instruments; however, the interpretation of this difference should be made cautiously, as minimal clinically important differences may vary according to patient population and clinical context.

In conclusion, MetS was associated with a higher pain burden in PsA and remained independently associated with higher pain severity after adjustment for disease activity and fibromyalgia, whereas its association with pain interference was not independent. Adiposity measures showed consistent independent associations, especially with interference, highlighting the potential relevance of metabolic and weight-related factors in shaping pain impact. These findings support a multidimensional approach to pain assessment in PsA that integrates disease activity with evaluation of metabolic comorbidity and pain amplification.

## Figures and Tables

**Table 1:** Demographic and clinical characteristics of the PsA study population.

Variable	Total (*n* = 187)	No MetS (*n* = 143)	MetS (*n* = 44)	*p*
Age	57 [51–63]	57 [49–62]	62 [54–65]	0.002
Female	137 (73.3)	105 (73.4)	32 (72.7)	0.9
BMI	26.6 [23.5–30.0]	25.5 [22.9–28.0]	30.1 [27.6–33.6]	<0.001
Overweight (BMI ≥ 25)	119 (63.6)	78 (54.5)	41 (93.2)	<0.001
Obesity (BMI ≥ 30)	47 (25.1)	22 (15.4)	25 (56.8)	<0.001
Hypertension	79 (42.5)	46 (31.9)	33 (78.6)	<0.001
Diabetes	41 (26.6)	15 (13.3)	26 (63.4)	<0.001
Disease duration	4.5 [2–9]	4 [2–8]	7 [3–12]	0.04
Conventional DMARD use	76 (40.6)	58 (40.6)	18 (40.9)	0.9
Biologic therapy use	152 (81.3)	117 (81.8)	35 (79.5)	0.7
Fibromyalgia	68 (36.4)	50 (35.0)	18 (40.9)	0.5
High triglycerides	27 (25.0)	4 (5.6)	23 (62.2)	<0.001
Low HDL	71 (59.2)	36 (45.0)	35 (87.5)	<0.001
Tender joint count (68)	1 [0–4]	1 [0–4]	0 [0–3]	0.2
Swollen joint count (66)	0 [0–0]	0 [0–0]	0 [0–0]	0.3
Leeds Enthesitis Index (LEI)	0 [0–0]	0 [0–0]	0 [0–0]	0.9
Dactylitis	5 (2.7)	4 (2.8)	1 (2.3)	0.7
PASI	0 [0–0]	0 [0–0]	0 [0–0]	0.4
CRP	0.20 [0.15–0.45]	0.20 [0.11–0.33]	0.28 [0.20–0.78]	0.02
ESR	11.1 [6–20]	10 [6–17.3]	16 [6–31.2]	0.03
DAPSA	14.1 [8.1–18.3]	14.1 [8.1–18.1]	14.4 [6.65–20.2]	0.8
Patient pain	6 [4–7]	6 [4–7]	7 [5–8.5]	0.1
Patient global assessment	5 [3–7]	5 [3–7]	6 [4–8]	0.2
Joint pain VAS	6 [4–8]	6 [4–8]	7 [5–8]	0.2
BPI severity	5.0 [2.75–6.75]	4.5 [2.5–6.5]	5.75 [3.38–7.12]	0.04
BPI interference	5.71 [2.71–7.43]	5.50 [2.57–7.00]	6.86 [3.46–8.00]	0.04
CSI total	50 [35–61]	49 [35–59]	52.5 [34.5–65.5]	0.4
HADS-Anxiety	7 [4–10]	7 [4–10]	7 [3.5–12]	0.7
HADS-Depression	7 [4–10]	7 [4–9]	8 [4.5–11]	0.2
HAQ-DI	0.88 [0.25–1.38]	0.75 [0.25–1.25]	1.25 [0.38–1.5]	0.03
PainDETECT	13 [7–20]	13 [7–20]	12.5 [6.5–22.5]	0.8
EQ-5D Mobility	1 [1–2]	1 [1–2]	2 [1–2]	0.01
EQ-5D Self-care	1 [0–1]	1 [0–1]	1 [0–2]	0.03
EQ-5D Usual activities	1 [1–2]	1 [1–2]	2 [1–3]	0.05
EQ-5D Pain/Discomfort	2 [1–2]	2 [1–2]	2 [2–3]	0.07
EQ-5D Anxiety/Depression	1 [0–2]	1 [0–2]	1 [0–2]	0.5
EQ-VAS	55 [50–70]	55 [50–70]	52.5 [42.5–70]	0.7

Legend: Continuous variables are reported as median [IQR]; categorical variables as *n* (%). *p* values refer to between-group comparisons (MetS vs. no MetS). Abbreviations: PsA, psoriatic arthritis; MetS, metabolic syndrome; BMI, body mass index; DMARD, disease-modifying antirheumatic drug; ; LEI, Leeds Enthesitis Index; PASI, Psoriasis Area and Severity Index; CRP, C-reactive protein; ESR, erythrocyte sedimentation rate; DAPSA, Disease Activity in Psoriatic Arthritis; VAS, visual analogue scale; BPI, Brief Pain Inventory; CSI, Central Sensitization Inventory; HADS, Hospital Anxiety and Depression Scale; HAQ-DI, Health Assessment Questionnaire–Disability Index; EQ-5D, EuroQol 5-dimension; EQ-VAS, EuroQol visual analogue scale.

**Table 2:** Univariable linear regression (BPI severity as dependent variable).

Variable	β Coefficient	95% CI	*p*
Age	−0.01	−0.04 to 0.03	0.7
Female	1.96	1.19 to 2.74	<0.001
BMI	0.06	−0.02 to 0.14	0.1
Overweight	0.20	−0.56 to 0.96	0.6
Obesity (BMI ≥ 30)	1.06	0.24 to 1.88	0.01
Disease duration	−0.05	−0.12 to 0.02	0.2
Conventional DMARD use	0.77	0.04 to 1.51	0.03
Biologic therapy use	0.53	−0.40 to 1.46	0.2
Fibromyalgia	2.40	1.73 to 3.07	<0.001
High triglycerides	0.11	−1.03 to 1.26	0.8
Low HDL	0.22	−0.71 to 1.15	0.6
Hypertension	0.27	−0.47 to 1.01	0.5
Diabetes	1.24	0.35 to 2.12	0.006
Metabolic syndrome	0.93	0.08 to 1.77	0.03
Tender joint count (68)	0.13	0.04 to 0.22	0.004
Swollen joint count (66)	0.39	−0.07 to 0.86	0.09
Leeds Enthesitis Index	0.31	−0.11 to 0.73	0.1
Dactylitis	−1.30	−2.79 to 0.20	0.08
PASI	0.11	−0.32 to 0.55	0.6
CRP	0.54	−0.18 to 1.27	0.1
ESR	0.03	0.00 to 0.05	0.01
DAPSA	0.16	0.12 to 0.20	<0.001
Patient pain	0.60	0.50 to 0.71	<0.001
Patient global assessment	0.61	0.51 to 0.71	<0.001
Joint pain VAS	0.84	0.75 to 0.94	<0.001
BPI interference	0.67	0.58 to 0.76	<0.001
CSI total	0.09	0.07 to 0.10	<0.001
HADS-Anxiety	0.17	0.09 to 0.25	<0.001
HADS-Depression	0.24	0.15 to 0.33	<0.001
HAQ-DI	1.96	1.48 to 2.43	<0.001
PainDETECT total	0.14	0.11 to 0.18	<0.001
EQ-5D Mobility	1.21	0.84 to 1.58	<0.001
EQ-5D Self-care	0.96	0.53 to 1.39	<0.001
EQ-5D Usual activities	1.07	0.75 to 1.39	<0.001
EQ-5D Pain/Discomfort	1.84	1.46 to 2.21	<0.001
EQ-5D Anxiety/Depression	0.54	0.17 to 0.92	0.005
EQ-VAS	−0.05	−0.06 to −0.03	<0.001

Legend: β, beta coefficient; CI, confidence interval; BPI, Brief Pain Inventory;BMI, body mass index; DMARD, disease-modifying antirheumatic drug; PASI, Psoriasis Area and Severity Index; CRP, C-reactive protein; ESR, erythrocyte sedimentation rate; DAPSA, Disease Activity in Psoriatic Arthritis; VAS, visual analogue scale; CSI, Central Sensitization Inventory; HADS, Hospital Anxiety and Depression Scale; HAQ-DI, Health Assessment Questionnaire–Disability Index; EQ-5D, EuroQol 5-dimension; EQ-VAS, EuroQol visual analogue scale.

**Table 3:** Univariable linear regression (BPI interference as dependent variable).

Variable	β Coefficient	95% CI	*p*
Age	−0.01	−0.05 to 0.03	0.7
Female	2.25	1.40 to 3.10	<0.001
BMI	0.08	−0.01 to 0.16	0.08
Overweight	0.40	−0.43 to 1.24	0.3
Obesity	1.46	0.56 to 2.36	0.002
Disease duration	−0.02	−0.10 to 0.06	0.6
Conventional DMARD use	0.56	−0.25 to 1.38	0.2
Biologic therapy use	0.45	−0.58 to 1.48	0.4
Fibromyalgia	2.43	1.67 to 3.19	<0.001
High triglycerides	0.06	−1.19 to 1.31	0.9
Low HDL	0.05	−0.97 to 1.07	0.9
Hypertension	0.24	−0.58 to 1.05	0.6
Diabetes	1.08	0.09 to 2.08	0.03
Metabolic syndrome	0.81	−0.13 to 1.75	0.09
Tender joint count (68)	0.08	−0.02 to 0.18	0.1
Swollen joint count (66)	0.38	−0.14 to 0.90	0.1
Leeds Enthesitis Index	0.40	−0.06 to 0.87	0.09
Dactylitis	−0.99	−2.65 to 0.67	0.2
PASI	−0.07	−0.55 to 0.41	0.8
CRP	0.97	0.18 to 1.77	0.01
ESR	0.03	0.01 to 0.06	0.01
DAPSA	0.15	0.10 to 0.19	<0.001
Patient pain	0.59	0.46 to 0.71	<0.001
Patient global assessment	0.55	0.43 to 0.68	<0.001
Joint pain VAS	0.73	0.59 to 0.87	<0.001
BPI severity	0.83	0.72 to 0.94	<0.001
CSI total	0.10	0.09 to 0.12	<0.001
HADS-Anxiety	0.32	0.23 to 0.40	<0.001
HADS-Depression	0.36	0.27 to 0.45	<0.001
HAQ-DI	2.48	1.99 to 2.98	<0.001
PainDETECT total	0.15	0.11 to 0.19	<0.001
EQ-5D Mobility	1.71	1.34 to 2.09	<0.001
EQ-5D Self-care	1.49	1.03 to 1.95	<0.001
EQ-5D Usual activities	1.54	1.21 to 1.86	<0.001
EQ-5D Pain/Discomfort	2.09	1.68 to 2.50	<0.001
EQ-5D Anxiety/Depression	1.22	0.83 to 1.60	<0.001
EQ-VAS	−0.06	−0.08 to −0.04	<0.001

Legend: β, beta coefficient; CI, confidence interval; BPI, Brief Pain Inventory; BMI, body mass index; DMARD, disease-modifying antirheumatic drug; PASI, Psoriasis Area and Severity Index; CRP, C-reactive protein; ESR, erythrocyte sedimentation rate; DAPSA, Disease Activity in Psoriatic Arthritis; VAS, visual analogue scale; CSI, Central Sensitization Inventory; HADS, Hospital Anxiety and Depression Scale; HAQ-DI, Health Assessment Questionnaire–Disability Index; EQ-5D, EuroQol 5-dimension; EQ-VAS, EuroQol visual analogue scale.

**Table 4:** Multivariable linear regression adjusted for age, sex, disease duration, fibromyalgia, and DAPSA (BPI severity as dependent variable).

Variable	β Coefficient	95% CI	*p*
Age	−0.01	−0.04 to 0.02	0.6
Female	0.83	0.09 to 1.57	0.03
Disease duration	−0.04	−0.10 to 0.02	0.1
Fibromyalgia	1.67	0.98 to 2.36	<0.001
Metabolic syndrome	0.99	0.23 to 1.75	0.01
DAPSA	0.11	0.07 to 0.16	<0.001

Legend: β, beta coefficient; CI, confidence interval; BPI, Brief Pain Inventory; DAPSA, Disease Activity in Psoriatic Arthritis.

**Table 5:** Multivariable linear regression adjusted for age, sex, disease duration, fibromyalgia, and DAPSA (BPI interference as dependent variable).

Variable	β Coefficient	95% CI	*p*
Age	−0.01	−0.04 to 0.03	0.6
Female	1.24	0.38 to 2.10	0.005
Disease duration	−0.01	−0.08 to 0.05	0.7
Fibromyalgia	1.65	0.85 to 2.45	<0.001
Metabolic syndrome	0.69	−0.20 to 1.57	0.1
DAPSA	0.09	0.04 to 0.14	<0.001

Legend: β, beta coefficient; CI, confidence interval; BPI, Brief Pain Inventory; DAPSA, Disease Activity in Psoriatic Arthritis.

## Data Availability

The data presented in this study are available on request from the corresponding author.The data are not publicly available due to privacy or ethical restrictions.

## Supplementary Materials

The following supporting information can be downloaded at: https://www.mdpi.com/article/10.3390/biomedicines14081752/s1.

## Abbreviations

The following abbreviations are used in this manuscript:

PsA	Psoriatic Arthritis
MetS	Metabolic Syndrome
BMI	Body Mass Index
BPI	Brief Pain Inventory
DAPSA	Disease Activity in Psoriatic Arthritis
CASPAR	Classification Criteria for Psoriatic Arthritis
ATP III	Adult Treatment Panel III
PROMs	Patient-Reported Outcome Measures
TJC	Tender Joint Count
SJC	Swollen Joint Count
PtGA	Patient Global Assessment
VAS	Visual Analogue Scale
PASI	Psoriasis Area and Severity Index
LEI	Leeds Enthesitis Index
CSI	Central Sensitization Inventory
painDETECT	PainDETECT Questionnaire
HADS	Hospital Anxiety and Depression Scale
HAQ	Health Assessment Questionnaire
HAQ-DI	Health Assessment Questionnaire–Disability Index
EQ-5D-5L	EuroQol 5-Dimension 5-Level Questionnaire
EQ-VAS	EuroQol Visual Analogue Scale
CRP	C-Reactive Protein
ESR	Erythrocyte Sedimentation Rate
DMARDs	Disease-Modifying Antirheumatic Drugs
csDMARDs	Conventional Synthetic Disease-Modifying Antirheumatic Drugs
bDMARDs	Biologic Disease-Modifying Antirheumatic Drugs
SD	Standard Deviation
IQR	Interquartile Range
CI	Confidence Interval
HRQoL	Health-Related Quality of Life
HDL	High-Density Lipoprotein
