# Supplementary material for: Metabolic Syndrome Is Independently Associated with Pain Severity in Psoriatic Arthritis
Source: Biomedicines. 2026 Aug 4;14(8):1752. doi: 10.3390/biomedicines14081752 (PMC13509873; doi:10.3390/biomedicines14081752)
Supplement: Supplementary file 1 [file biomedicines-14-01752-s001.zip › biomedicines-4370997-supplementary.pdf]

Supplementary Table S1. Multivariable linear regression adjusted for BMI, age, sex, disease duration, fibromyalgia, and DAPSA (BPI severity as dependent variable)

Variable	β coefficient	95% CI	p
**Age**	-0.01	-0.04 to 0.02	0.703
**Female**	0.91	0.16 to 1.66	0.018
**Disease duration**	-0.04	-0.10 to 0.02	0.189
**Fibromyalgia**	1.79	1.10 to 2.48	<0.001
**BMI**	0.10	0.03 to 0.16	0.004
**DAPSA**	0.12	0.07 to 0.16	<0.001

Legend: β, beta coefficient; CI, confidence interval; BMI, body mass index; DAPSA, Disease Activity in Psoriatic Arthritis; BPI, Brief Pain Inventory. Obesity was defined as BMI ≥30 kg/m²; overweight as BMI ≥25 kg/m².

Supplementary Table S2. Multivariable linear regression adjusted for obesity, age, sex, disease duration, fibromyalgia, and DAPSA (BPI severity as dependent variable)

Variable	β coefficient	95% CI	P
**Age**	0.00	-0.03 to 0.03	0.887
**Female**	0.77	0.03 to 1.51	0.041
**Disease duration**	-0.04	-0.10 to 0.02	0.224
**Fibromyalgia**	1.78	1.09 to 2.47	<0.001
**Obesity**	0.91	0.22 to 1.60	0.010
**DAPSA**	0.11	0.07 to 0.15	<0.001

Legend: β, beta coefficient; CI, confidence interval; BMI, body mass index; DAPSA, Disease Activity in Psoriatic Arthritis; BPI, Brief Pain Inventory. Obesity was defined as BMI ≥30 kg/m²; overweight as BMI ≥25 kg/m².

Supplementary Table S3. Multivariable linear regression adjusted for overweight, age, sex, disease duration, fibromyalgia, and DAPSA (BPI severity as dependent variable)

Variable	β coefficient	95% CI	P

**Age**	0.00	-0.03 to 0.03	0.789
**Female**	0.86	0.11 to 1.62	0.025
**Disease duration**	-0.04	-0.10 to 0.02	0.190
**Fibromyalgia**	1.82	1.12 to 2.52	<0.001
**Overweight**	0.65	-0.01 to 1.30	0.052
**DAPSA**	0.11	0.07 to 0.15	<0.001

Legend: β, beta coefficient; CI, confidence interval; BMI, body mass index; DAPSA, Disease Activity in Psoriatic Arthritis; BPI, Brief Pain Inventory. Obesity was defined as BMI ≥30 kg/m²; overweight as BMI ≥25 kg/m².

Supplementary Table S4. Multivariable linear regression adjusted for diabetes, age, sex, disease duration, fibromyalgia, and DAPSA (BPI severity as dependent variable)

Variable	β coefficient	95% CI	P
**Age**	-0.01	-0.04 to 0.2	0.7
**Female**	0.6	-0.11 to 1.49	0.1
**Disease duration**	-0.01	-0.08 to 0.06	0.2
**Fibromyalgia**	1.81	1.04 to 2.59	<0.001
**Diabetes**	1.1	0.35 to 1.97	0.005
**DAPSA**	0.11	0.06 to 0.15	<0.001

Supplementary Table S5. Multivariable linear regression adjusted for BMI, age, sex, disease duration, fibromyalgia, and DAPSA (BPI interference as dependent variable)

Variable	β coefficient	95% CI	P
**Age**	-0.01	-0.04 to 0.03	0.657
**Female**	1.39	0.52 to 2.25	0.002
**Disease duration**	-0.01	-0.08 to 0.06	0.730

**Fibromyalgia**	1.74	0.95 to 2.53	<0.001
**BMI**	0.10	0.02 to 0.17	0.015
**DAPSA**	0.09	0.04 to 0.14	<0.001

Legend: β, beta coefficient; CI, confidence interval; BMI, body mass index; DAPSA, Disease Activity in Psoriatic Arthritis; BPI, Brief Pain Inventory. Obesity was defined as BMI ≥30 kg/m^2; overweight as BMI ≥25 kg/m^2.

Supplementary Table S6. Multivariable linear regression adjusted for obesity, age, sex, disease duration, fibromyalgia, and DAPSA (BPI interference as dependent variable)

**Variable**	**β coefficient**	**95% CI**	**P**
**Age**	-0.01	-0.04 to 0.03	0.769
**Female**	1.20	0.36 to 2.05	0.005
**Disease duration**	-0.01	-0.08 to 0.06	0.751
**Fibromyalgia**	1.74	0.96 to 2.53	<0.001
**Obesity**	1.22	0.43 to 2.01	0.003
**DAPSA**	0.09	0.04 to 0.13	0.001

Legend: β, beta coefficient; CI, confidence interval; BMI, body mass index; DAPSA, Disease Activity in Psoriatic Arthritis; BPI, Brief Pain Inventory. Obesity was defined as BMI ≥30 kg/m^2; overweight as BMI ≥25 kg/m^2.

Supplementary Table S7. Multivariable linear regression adjusted for overweight, age, sex, disease duration, fibromyalgia, and DAPSA (BPI interference as dependent variable)

**Variable**	**β coefficient**	**95% CI**	**P**
**Age**	-0.01	-0.04 to 0.03	0.721
**Female**	1.30	0.43 to 2.16	0.003
**Disease duration**	-0.01	-0.08 to 0.05	0.680
**Fibromyalgia**	1.77	0.97 to 2.57	<0.001
**Overweight**	0.67	-0.08 to 1.41	0.081

**DAPSA**	0.09	0.04 to 0.14	0.001

Legend: β, beta coefficient; CI, confidence interval; BMI, body mass index; DAPSA, Disease Activity in Psoriatic Arthritis; BPI, Brief Pain Inventory. Obesity was defined as BMI ≥30 kg/m²; overweight as BMI ≥25 kg/m².
